# Opportunities and Challenges for Alternative Nanoplasmonic Metals: Magnesium and Beyond

**DOI:** 10.1021/acs.jpcc.2c01944

**Published:** 2022-06-23

**Authors:** Elizabeth R. Hopper, Christina Boukouvala, Jérémie Asselin, John S. Biggins, Emilie Ringe

**Affiliations:** † Department of Materials Science and Metallurgy, University of Cambridge, 27 Charles Babbage Road, Cambridge CB3 0FS, United Kingdom; ‡ Department of Earth Sciences, University of Cambridge, Downing Street, Cambridge CB2 3EQ, United Kingdom; § Department of Chemical Engineering and Biotechnology, University of Cambridge, Philippa Fawcett Drive, Cambridge CB3 0AS, United Kingdom; ∥ Department of Engineering, University of Cambridge, Trumpington Street, Cambridge CB2 1PZ, United Kingdom

## Abstract

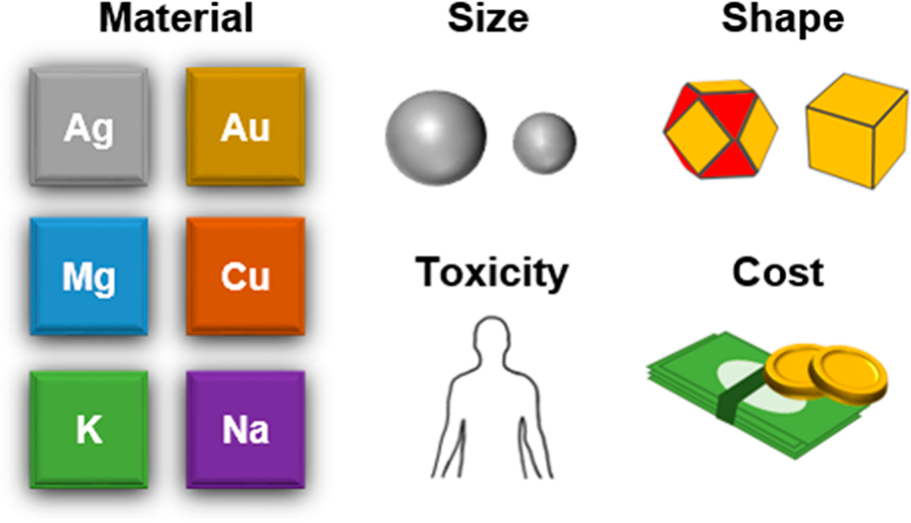

Materials that sustain localized surface plasmon resonances have a broad technology potential as attractive platforms for surface-enhanced spectroscopies, chemical and biological sensing, light-driven catalysis, hyperthermal cancer therapy, waveguides, and so on. Most plasmonic nanoparticles studied to date are composed of either Ag or Au, for which a vast array of synthetic approaches are available, leading to controllable size and shape. However, recently, alternative materials capable of generating plasmonically enhanced light–matter interactions have gained prominence, notably Cu, Al, In, and Mg. In this Perspective, we give an overview of the attributes of plasmonic nanostructures that lead to their potential use and how their performance is dictated by the choice of plasmonic material, emphasizing the similarities and differences between traditional and emerging plasmonic compositions. First, we discuss the materials limitation encapsulated by the dielectric function. Then, we evaluate how size and shape maneuver localized surface plasmon resonance (LSPR) energy and field distribution and address how this impacts applications. Next, biocompatibility, reactivity, and cost, all key differences underlying the potential of non-noble metals, are highlighted. We find that metals beyond Ag and Au are of competitive plasmonic quality. We argue that by thinking outside of the box, i.e., by looking at nonconventional materials such as Mg, one can broaden the frequency range and, more importantly, combine the plasmonic response with other properties essential for the implementation of plasmonic technologies.

## Introduction

Small is different. At the nanoscale (1–100 nm), surface dominates, dramatically affecting the behavior of materials. A striking example is Au: inert, yellow, and shiny in the bulk, it becomes a brightly colored catalyst when made into nanoparticles (NPs).^1,2^ Since its synthesis by Faraday in the 19th century,^3,4^ colloidal Au has gained much attention, in particular owing to its optical properties. A limited number of other metals, including Al, Cu, Mg, In, and Ag, have, over the past decades, demonstrated interactions with light similar to that of Au’s, while offering alternative chemical, biological, and catalytic properties.^5−7^ In this Perspective, we use Mg as an example to describe how expanding the nanotechnology toolbox with metals beyond Ag and Au leads to new opportunities and enhanced tunability.

The optical properties of nanoscale Au result from localized surface plasmon resonances (LSPRs).^1^ Simply put, an electric field can polarize the electron cloud in metallic NPs, and upon its removal, restoring Coulomb forces from the positively charged nuclei move the electrons back in the opposite direction, with some inertia leading to a natural oscillator akin to a mass on a spring.^8^ If the incident electric field is oscillating, such as that of light, this nanoscale oscillator can be driven into resonance. The resonant frequency depends not only on the properties of the electron cloud and underlying lattice (composition) but also on the surrounding medium as well as the NP’s size and shape. Another, equally valid, way to describe LSPRs is that they are surface plasmons, i.e., charge oscillations at a metal–dielectric interface, confined to finite, nanoscale surfaces with a characteristic size and shape.^9^ They dominate optical properties in NPs, e.g., colloidal Au, because there is so much surface.

A material can sustain LSPRs if it has sufficiently polarizable electrons and low losses. More specifically, resonances of high amplitude can exist at frequencies where the dielectric function of a material has a large negative real and a small positive imaginary component.^6^ This occurs in or around the visible range of electromagnetic frequencies for only a few metals, the most common being Ag and Au.

When a suitable material is excited, LSPRs produce vibrant colors resulting from their wavelength-dependent photon absorption and scattering. These are called far-field effects as they are observed away from the NP. Another effect, now in the near-field, is a local enhancement of the incident electric field at the surface of the NP. The quite rapid decay of LSPRs also produces excited charge carriers and eventually heat.^10^

Far-field and near-field effects have been investigated for a myriad of potential sensing applications. The NP’s far-field response depends on its surrounding dielectric environment, leading to sensing capabilities; yet, the longer established SPR-based technologies are dominant over LSPR approaches for such sensing applications, in particular for biomedical research. The local electric field enhancement increases the signal of optical spectroscopies, enabling techniques such as surface-enhanced Raman scattering (SERS),^11,12^ surface-enhanced infrared spectroscopy (SEIRA),^13^ and metal-enhanced fluorescence (MEF).^14,15^ These analytical tools are key to scientific research and enabled discoveries in biology, catalysis, and chemistry to name a few.

Recently, plasmon decay products, namely heat and excited charge carriers, have attracted much attention. Indeed, as the absorption cross-section of plasmonic particles can be greater than their physical cross-section, they can act as antennae for light, capturing light’s energy which could then be used to power processes. A multitude of plasmonic metals have been demonstrated to drive chemical reactions, produce local heating, or both.^10,16,17^ While the use and effect of “hot” electrons and holes remains debated, numerous reports of reaction enhancement and changes in selectivity exist,^10,18^ securing a convincing potential function for plasmonics as a light-capturing element for chemical reactions. A promising approach for such applications is to coat a rather large (∼100 nm), thus efficiently light-capturing, plasmonic structure with very small amounts of catalytically active materials that act as a catalyst surface. In this “antenna–reactor” construct,^19^ the volume of plasmonic metal exceeds that of the catalyst by roughly 2 orders of magnitude, putting pressure on the cost of the former but much less on that of the latter. Further cost efficiency would come from using the sun as the light source, hence the growing interest in materials with absorption well-matched with the solar spectrum. Another application of plasmon decay is their use as photothermal nanomaterials. Heating effects are well-established, especially for Au. Au NPs have indeed been shown to boil water^20^ and kill cancer cells via hyperthermia *in vitro* and *in vivo* using near-infrared (NIR) illumination, leading to many clinical trials, with some NPs on the path to approval.^21,22^ In this case, resonances in the NIR are preferred in order to utilize biological transparency windows.

Historically, plasmonic materials have been Au and Ag; they are relatively simple to synthesize and sustain LSPRs in the visible range. Recently, the interest in nanoscale control of light has led to the study of an increasingly high number of new plasmonic materials, for instance metal nitrides,^23^ graphene,^24^ and superconductors.^25^ These have been reviewed by other authors, including Tassin *et al.* who concluded that the performance of metals is generally compelling in and around the visible range.^26^ Meanwhile, doped metal oxides such as indium tin oxide (ITO)^27^ and fluorine/indium cadmium oxide (FICO)^28^ as well as metal chalcogenides of varied stoichiometry^36^ have emerged as widely tunable platforms for plasmonics. These are also reviewed elsewhere,^29−36^ and in this Perspective, we will focus on comparing the performance and properties of metallic structures. Of the metals, several alternatives to Ag and Au have emerged over the past decades. Historically, composition (and plasmon) tuning has been achieved by alloying, mostly of Ag and Au (but also Cu);^37−40^ more recently, alternative metals have been explored, including Cu, Al, In, and Mg. These metals sustain plasmonic behavior and, more importantly in our opinion, a range of other desirable properties such as low cost, biocompatibility, different plasmonic ranges, and chemical reactivity.

In this Perspective, we provide an overview of the attributes of plasmonic structures that lead to their potential use and discuss the current state of the field. We argue that, by thinking outside of the box, i.e., by looking at nonconventional materials such as Mg, it is possible to combine the plasmonic response with other key properties essential to broadening the implementation of plasmonic technologies.

This Perspective is organized in sections covering the fundamental performance limits encoded in the dielectric function; the effect of size and shape in the tuning of near- and far-field plasmonic properties; material consideration in biocompatibility; reactivity and oxidation as a challenge and an opportunity; finally, costs in various contexts. For each section, the basic scientific concepts are reviewed, followed by a critical assessment of the current state-of-the-art and proposed ways forward. We conclude that not all aspects of nanoplasmonic science are limiting factors to the establishment of viable commercial applications and that alternative metals offer suitable plasmonic performance with the potential for new applications *via* their low cost, high biocompatibility, and different chemistry.

## Discussion

### The Dielectric Function Shapes LSPRs

The starting point for a comparison of nanoplasmonic materials must be quantifying how good a plasma they contain. Generically, a plasma is a gas-like assembly of free charges, which move following inertial dynamics.^8^ Traditional plasmas are ionized gases, and they are found at astrophysical scales, in the ionosphere, and within fluorescent light bulbs. However, perhaps surprisingly, the conduction electrons within metals are also sufficiently free to form a plasma, particularly in alkali and noble metals where a free-electron gas model is extremely predictive of electronic properties.^8^ Importantly, all plasmas are natural oscillators: if the cloud of free electrons is displaced and released, it will bounce backward and forward much like a mass on a spring due to the combination of a restoring Coulomb force (from the immobile ions) and the electron inertia. These bulk charge oscillations occur at a characteristic plasma frequency (ω_p_), which, for an idealized free-electron gas, depends on the number density of electrons (*n*) and the mass of an electron (*m*):^9^
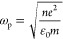
1Such oscillations will be driven to high amplitude if excited by light at ω_p_, a phenomenon known as bulk plasmon resonance. In real plasmas, damping of the charge’s motion limits the amplitude of this resonance, and the degree of damping encodes the quality of the plasma.

**Figure 1:**
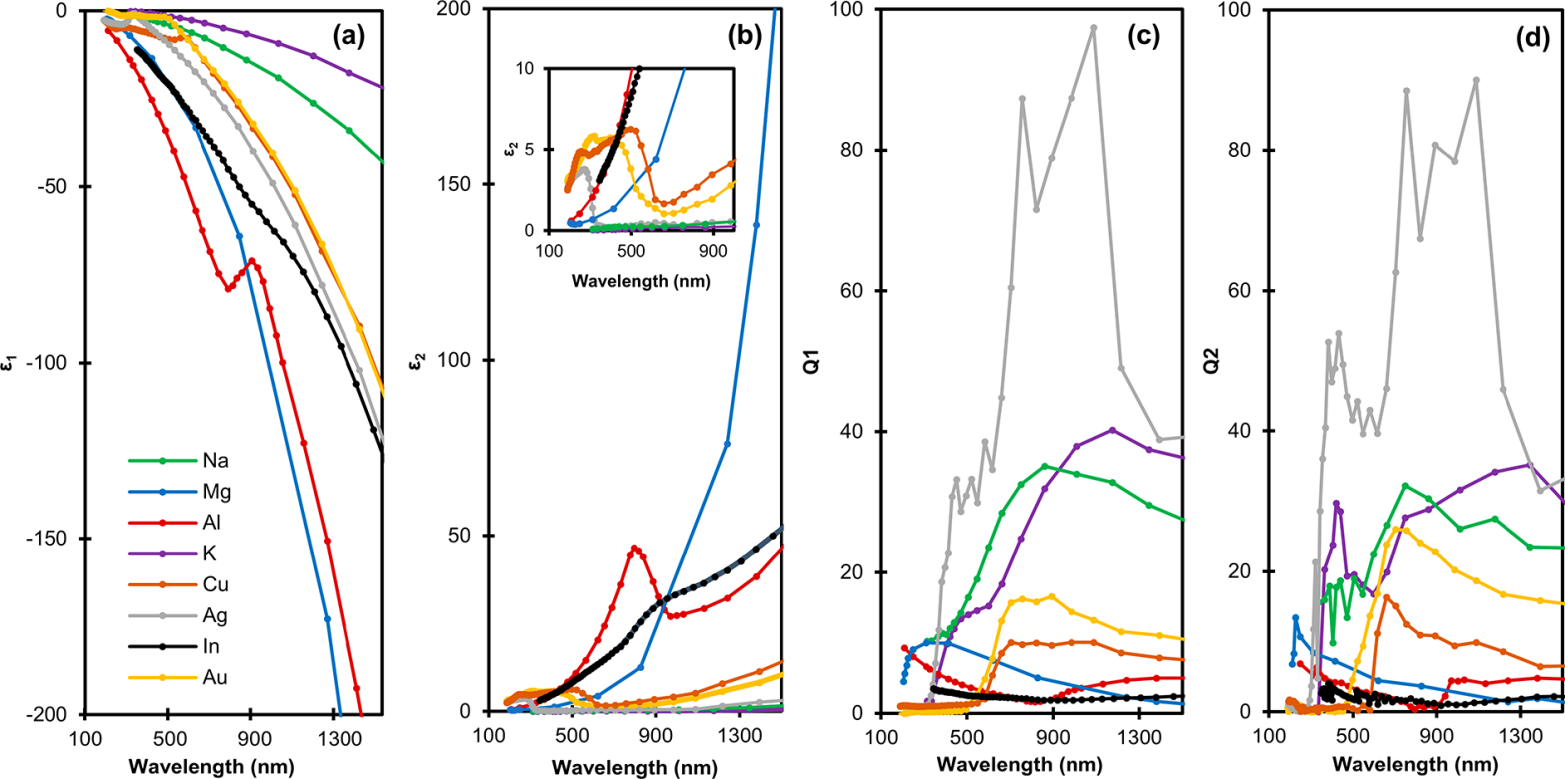
(a) Real and (b) imaginary part of the dielectric function of various plasmonic metals with the corresponding merit indices (c) Q1 and (d) Q2. Values were obtained from Johnson and Christy^44^ (Au, Ag, Cu), Palik^45^ (Mg), Rakić^46^ (Al), Smith^47^ (Na, K), and Mathewson and Myers^48^ (In).

In general, the response of a material to an oscillating electric field (i.e., light) is captured by its complex dielectric function ε(ω) (Figure 1):
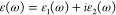
2Both ideal plasma behavior and damping are encoded in a plasma’s dielectric function. An idealized free-electron treatment of a plasma gives a purely real dielectric function (with a strong frequency dependence) that changes from negative to positive at ω_p_:
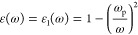
3More sophisticated material-specific models can be formulated to account for collisions, residual polarization due to the positive ion cores, and damping of the charge’s motion *via*, for example, interband transitions. These models all lead to the same basic behavior in which ε_1_(ω) changes sign from negative to positive at the plasma frequency but also include a finite ε_2_(ω) associated with damping and which ultimately limits the material’s plasmonic performance.

The sign change in ε_1_ is also associated with the transition from reflective to transmissive for bulk metals, meaning that below ω_p_, light cannot penetrate bulk metal at all. However, even below ω_p_, one can still have natural charge oscillations at the interface between a plasmonic metal and an insulator, which give rise to propagating SPRs. When these surface oscillations are confined to the surface of a NP, they are discretized into standing-wave like modes (LSPRs) with resonant frequencies well below ω_p_. The simplest case of such an LSPR is seen in the polarizability (α) of a small spherical NP of radius *a* embedded in a medium with a real, positive dielectric constant ε_m_:
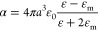
4In an idealized lossless plasma (ε_2_ = 0), this polarizability would diverge at the Fröhlich condition, ε_1_ = −2ε_m_, indicating the dipole LSPR frequency of the sphere. As expected, the resonance occurs in the negative region of ε_1_, below the plasma frequency: for a material exactly following eq 2, it would be at . In a real material, the resonant polarization will be proportional to 1/ε_2_, which, though not infinite, can be very large if losses are small. Higher order modes and nonspherical particles behave similarly but with different critical values for ε_1_ and, hence, different resonant frequencies.

Accordingly, a good LSPR material needs an ε_1_ that spans a large range of negative values so that many resonance criteria (for many different surrounding environments) can be hit, and also needs a small ε_2_ so that damping is low and resonances are strong. In this limit of low damping and strong resonances, the resonant amplitude given by eq 4 is simply proportional to
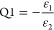
5and the same ratio also occurs in many other geometries, leading it to be proposed by Arnold and Blaber^41^ as a universal figure of merit for LSPR material selection. An alternative figure of merit is the quality factor of an LSP mode which, like for any resonant oscillator, is defined as the ratio of the resonant frequency to the bandwidth of the resonance or, equivalently, as the number of radians of free oscillation for the stored energy to decay by 1/*e*. In principle, the quality factor of an LSPR would depend on the details of the particle and mode shape; however, at least in the small-particle (electrostatic) limit, there is a generic argument that it actually depends solely on the dielectric function at the resonant frequency and is given by^42,43^
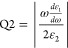
6

The real and imaginary part of the dielectric function for several plasmonic metals is plotted in Figure 1, along with Q1 and Q2. Elements with plasmonic behavior include the alkali metals (Na, K), Mg, and the noble metals (Cu, Ag, Au) as well metals in the boron group (Al, Ga, In). When one looks at either the loss profile, Q1, or Q2, similar trends in the performance of metals across the UV, visible, and NIR are revealed. The starkest observation is that none of the metals’ quality factors (Q2) are very high, indicating that NP LSPRs are always rather lossy. The standout performer is Ag with quality factors rising near 100 in the visible range, far above the competition. However, despite dominating the field, Au is not particularly impressive, and several other candidates offer equal or better performance, while also being cheaper and possessing different chemical profiles. In particular, Mg and Al, both non-noble metals, have higher Q1 in the blue and UV regions than Cu and Au, both noble metals. However, the plasmonic behavior is not simply a function of noble vs non-noble metals: Na and K, for instance, track the performance of Ag.

In more detail, below 300 nm, Mg, Na, and K are actually the best candidates (highest Q1 and Q2), followed closely by Al and with both Ag and Au somewhat behind. This high-frequency performance can be attributed to the free-electron nature of the alkali metals and Mg. Ag performs quite badly in this region, owing to its d-orbital interband transitions which are naturally absent in the s-valent alkali and alkali earth elements; for instance, at 310 nm, Q1 for Ag is 1.2, while it is 10.3, 1.7, and 10.0 for Na, K, and Mg, respectively.^44,45,47^ In the visible range, Ag performs best and Q1 and Q2 paint slightly different pictures of the runners up with Q2 indicating a close match between Na, K, and Au, while Q1 favors Na and K (Figure 1). An explicit comparison of the emerging plasmonic candidates Al, In, and Mg shows that Mg outperforms Al and In throughout the range of 300–900 nm (even if one uses the more lossy dielectric function reported by Palm *et al*.^49^) and highlights that Al performs particularly poorly in the NIR, again owing to interband transitions occurring at ∼800 nm. It is also noteworthy that the real part of the dielectric functions of Mg and Al exhibit the steepest and most negative values (Figure 1a) of all the candidates, potentially allowing them to exhibit the widest range of modes in the widest range of environments.

The descriptors mentioned above highlight the dependence of plasmonic performance on the material’s dielectric function. The wide variety of plasmonic applications lead to the need for a wide variety of plasmon energy, achievable through different compositions. For instance, visible light is used for solar-driven chemical reactions^19^ and NIR, in biomedical applications where biological transparency windows can be exploited. Meanwhile, in MEF, plasmon peaks in the UV can enhance the fluorescent signal of biomolecules that occurs naturally at such wavelengths.^50,51^ The expansion of the plasmonics toolbox beyond elemental metals can also optimize optical properties via material design. The inverse relationship of the imaginary part in Q1 and Q2 suggests that minimizing losses or shifting them away from the spectral region of interest will lead to a better plasmonic performance.^52^ One approach for such loss minimization is to engineer the band structure via alloying; however, most alloys, intermetallics, and silicides, either binary or tertiary, still suffer from interband transitions.^53^ The commonly observed monotonic relationship between the alloying materials and the dielectric function means alloying does not always promise an improved plasmonic response. Excitingly, not all alloys behave monotonically: some element combinations, such as the nonisovalent alloys of Au and Pd,^54^ display sensitive nonmonotonic relationships between the alloying concentration and the dielectric function with the function in some cases exceeding that of either of the constituents. Furthermore, alloying can enhance other properties such as corrosion and degradation.^55,56^ Rationally combining elements for stability and plasmonic response may be a way to stabilize the reactive group I metals and instill some of their low losses and high quality resonances into another metal; such promising nanostructures have yet to be reported. Overall, while there is a limited track record of drastic improvements in plasmonic quality due to the fundamental nature of the materials, a new discovery that would dramatically change the quality factor has the potential to propel plasmonics to a new era.

### Size and Shape Tune Plasmon Energy and Properties

The Fröhlich condition would suggest that LSPR frequencies are independent of NP size, giving little control over resonant frequency without altering the underlying material. However, this conclusion fortunately only applies to particles that are much smaller than the wavelength of light, which can be treated in the electrostatic limit. For NPs comparable in size to the wavelength, in practice this means ∼50 nm and above, size and shape can be used to manipulate LSPRs.^57^ This tuning allows navigation through the range of available wavelengths for each material^58^ as well as manipulation of the electric field distribution (near-field); ultimately, these properties dictate performance. Therefore, for any application, some optimization of size, shape, and material is needed to get the required characteristics at the operating wavelength.^59,60^ The control of NP size and shape has thus attracted much research effort and is now a well-established field: for all compositions, several size control strategies exist, while for the best known face-centered cubic (FCC) metals (Au, Ag),^61−66^ a vast library of shapes is achievable and is in the process of being recreated for the other FCC plasmonic materials (Al, Cu).^67−75^ Meanwhile, a new set of shapes with potentially competitive performance is emerging due to the different crystal structure of Mg, which is hexagonal close-packed (HCP).^76^

Fabrication techniques like lithography allow arbitrary control over size and shape.^77^ Correspondingly, much of the early, fundamental work was on fabricated structures, first for Au, and now for the emerging materials. However, these methods produce low quantities and poor crystallinity.^78^ Impactful applications are likely to require large quantities of highly crystalline NPs with low costs as well as a decent size and shape control. Solution-phase colloidal syntheses of metal NPs are inherently easier to scale-up, either in batch (by increasing the reactor size) or in continuous flow (by running reactions in parallel). For Au and Ag, colloidal syntheses are well-established and promising colloidal approaches are now emerging for the other plasmonic metals, enabling their recent study. This section on size and shape effects will therefore exclusively draw examples from colloidal syntheses. Such colloidal approaches are often simple aqueous reactions for Ag and Au and involve the air- and water-free toolkits of organic chemistry for the more reactive Al and Mg.^67,79^

**Figure 2:**
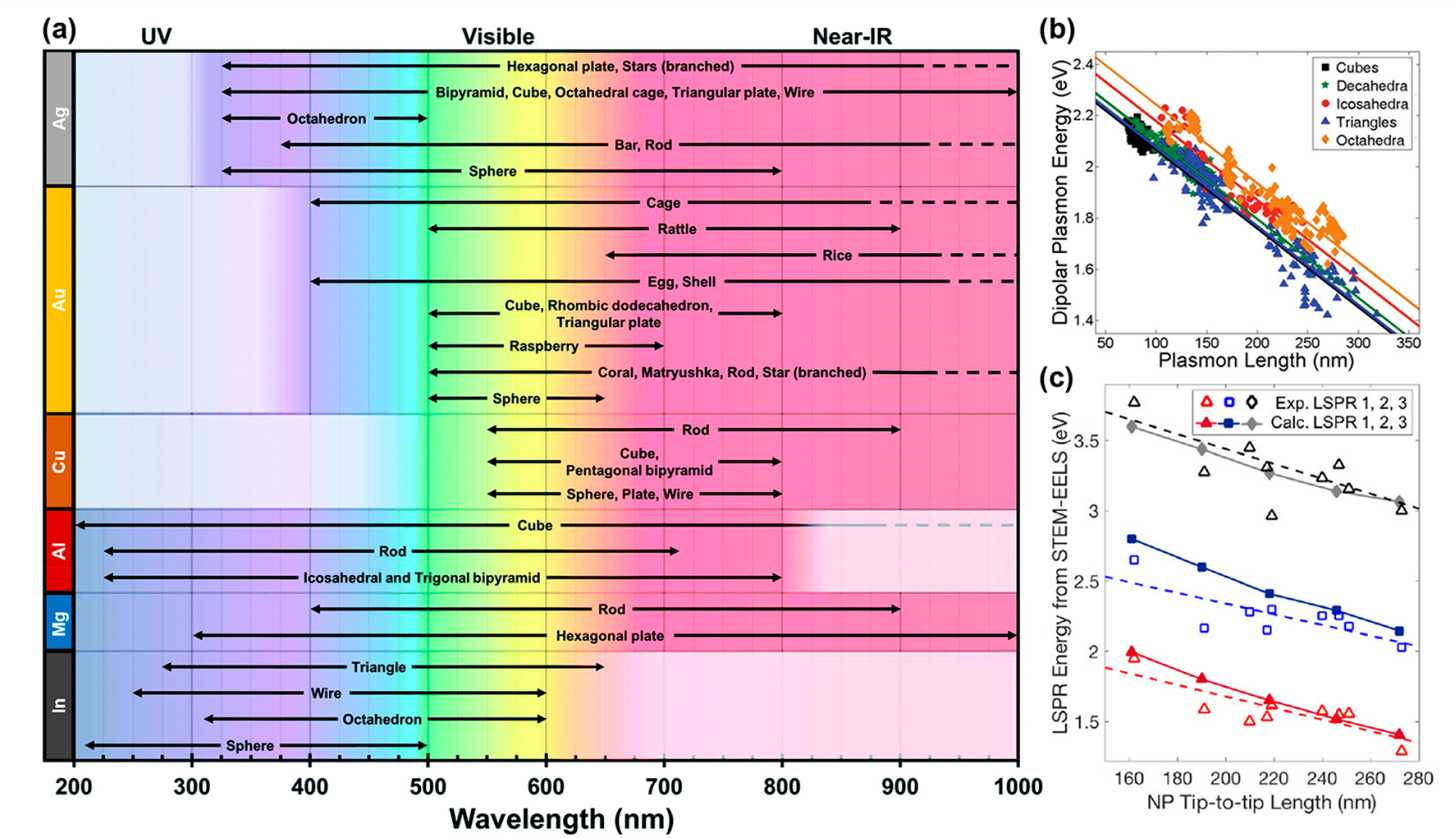
LSPR size and shape control. (a) Plasmonic spectral range of Ag, Au, Cu, Al, Mg, and In NPs over the UV–vis–NIR spectrum for various NP shapes. Paler colors indicate intrinsically low plasmonic performance regions due to interband transitions, and dashed lines indicate a potential extension of LSPR frequency in the NIR. Adapted from ref (80) with permission from the Royal Society of Chemistry. (b) Dipolar plasmon energy dependence on the plasmon length of Au NPs with different geometries. Reprinted from ref (58). Copyright 2012 American Chemical Society. (c) Experimental (open markers, dashed lines) and calculated (filled markers, solid lines) size dependence of the three lowest energy LSPRs observed in hexagonal Mg NPs. Reprinted from ref (79). Copyright 2018 American Chemical Society.

Size is a powerful means to manipulate LSPR frequency. With Au and Ag, size has been used to tune LSPR frequency across their entire plasmonic range (Figure 2a). With new materials comes the promise of an expanded operating range, contingent on the development of appropriate synthetic approaches. Great progress has been made on this front for various shapes and compositions, including recently on alternatives to Ag and Au, as shown in Figure 2a. Size effects arise because phase retardation, a phenomenon observed when the particle size is no longer negligible compared to the radiation wavelength, leads to a redshift of the resonance frequency proportional to the size. This shift becomes independent of shape for a given material (Figure 2b) if the size is expressed as plasmon length, defined as the length over which the plasmon oscillation takes place.^58^ As size increases, higher order resonances appear at higher energies, still within the plasmonic range of the material, which enable resonances over a broad wavelength range in a single particle, as shown in Figure 2c for Mg. Neither phase retardation nor higher order modes are included in the simple merit indices discussed earlier; in reality, each size and each mode has its own quality factor. As such, the materials’ figures of merit are simply a starting point in the search for high quality resonances, and modes being discovered in new plasmonic nanocrystals have the potential for attractive qualities across an expanded wavelength range.

Practically, in colloidal syntheses, NP size is dictated by the rate of nucleation, rate of growth, and growth time. Approaches to controlling the rates and duration of these steps include changing precursor concentration as well as manipulating reduction potentials and temperature. Such approaches have been widely successful for well-known plasmonic metals, as exemplified by the range of LSPR energies achieved for the shapes shown in Figure 2a. We recently demonstrated the tuning of Mg NP size between below 50 nm and up to over 1000 nm,^81^ and similar works have been published for Cu and Al,^70,72^ showing size tuning feasibility. Overall, good size control can be obtained for both established and emerging plasmonic metals with some work remaining to be done in narrowing the size distribution.

Shape is a powerful tool to control the field distribution and plasmon modes sustained by a NP. Shape anisotropy allows one to tweak the LSPR and lift the degeneracy of modes along various directions; for instance, the dipole of a sphere becomes a transverse and a longitudinal dipole in a rod. These modes display distinct energies and field distributions.^58,82^ With increasingly complex shapes, increasingly complex patterns develop, and they are further enriched by the appearance of higher order modes in large nanostructures.

Differently shaped NPs lead to plasmon modes with wildly different field enhancements, as captured by the Faraday number:^83^
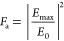
7where *E*_max_ is the maximum electric field amplitude and *E*_0_, the incoming electric field amplitude. The dramatic field enhancement of LSPRs underpins their great promise for the first application we will consider here: field-enhanced spectroscopies such as SERS, SEIRA, and MEF. For example, the degree of SERS enhancement is essentially *F*_a_^2^. Like for the LSPR frequency, simple approximations of the Faraday number for spheres exist to compare compositions (highlighting the excellent performance of Mg in the UV^60^) but do not provide much guidance in the case of realistic shapes. Moreover, shape really matters for field distribution, which tends to greatly concentrate near sharp features: for instance, the field intensity is much larger at the tips of a nanorod excited at its longitudinal dipole resonance than on the side of a sphere excited at its dipole,^82^ which is reflected in an ∼10 times higher SERS enhancement for Au.^84^ This tip light confinement is even more intense in NPs with sharper features such as the spikes of nanostars, leading to a potentially higher SERS enhancement.^85^

Another plasmonic application is refractive index sensing for which the figure of merit (FOM_LSPR_) is reported as the LSPR peak shift per refractive index unit over the resonance line width.^86^ This figure of merit is strongly influenced by shape as well as size and composition. Again, some insight can be provided by the electrostatic-limit FOM_LSPR_, which is simply proportional to Q1 with the inclusion of the refractive index of the surrounding medium η:^87^
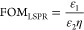
8Experimental evidence indeed indicates that Ag outperforms Au,^88^ as predicted by eq 8. Accordingly, the performance of any new material will also follow Q1. However, this material-based figure of merit does not take shape and size into account. Here, sharp tips also improve performance, as does having low energy modes with minimal overlap with other modes and narrow resonances.^89^ For instance, the FOM_LSPR_ of 15 nm Au spheres is 0.6, while it is 2.6 for Au nanorods (40 nm long, 17 nm wide) and 4.5 for 189 nm long, 40 nm wide Au nanobipyramids (essentially sharpened rods),^90^ an order of magnitude improvement achieved by shape optimization.

Finally, photothermal effects are at the basis of another set of plasmonic NP applications. A suitable material-related figure of merit is the heat power delivered, which has been studied for multiple metal spheres.^60^ This and the temperature increase observed depend on the absorption cross-section, which is also size and shape dependent.^91^ For instance, ref (92) describes how the photothermal transduction efficiency is over two times lower in Au nanoshells than in Au nanorods. Generally, as size increases, both the absolute absorption and the scattering to absorption ratio increase, such that there tends to be an “ideal” size for each shape.^93^ The shape can then be used to navigate the plasmon resonance and performance to match the desired illumination, *i.e.*, in the NIR region for biological applications and the visible region for sunlight-driven systems.

**Figure 3:**
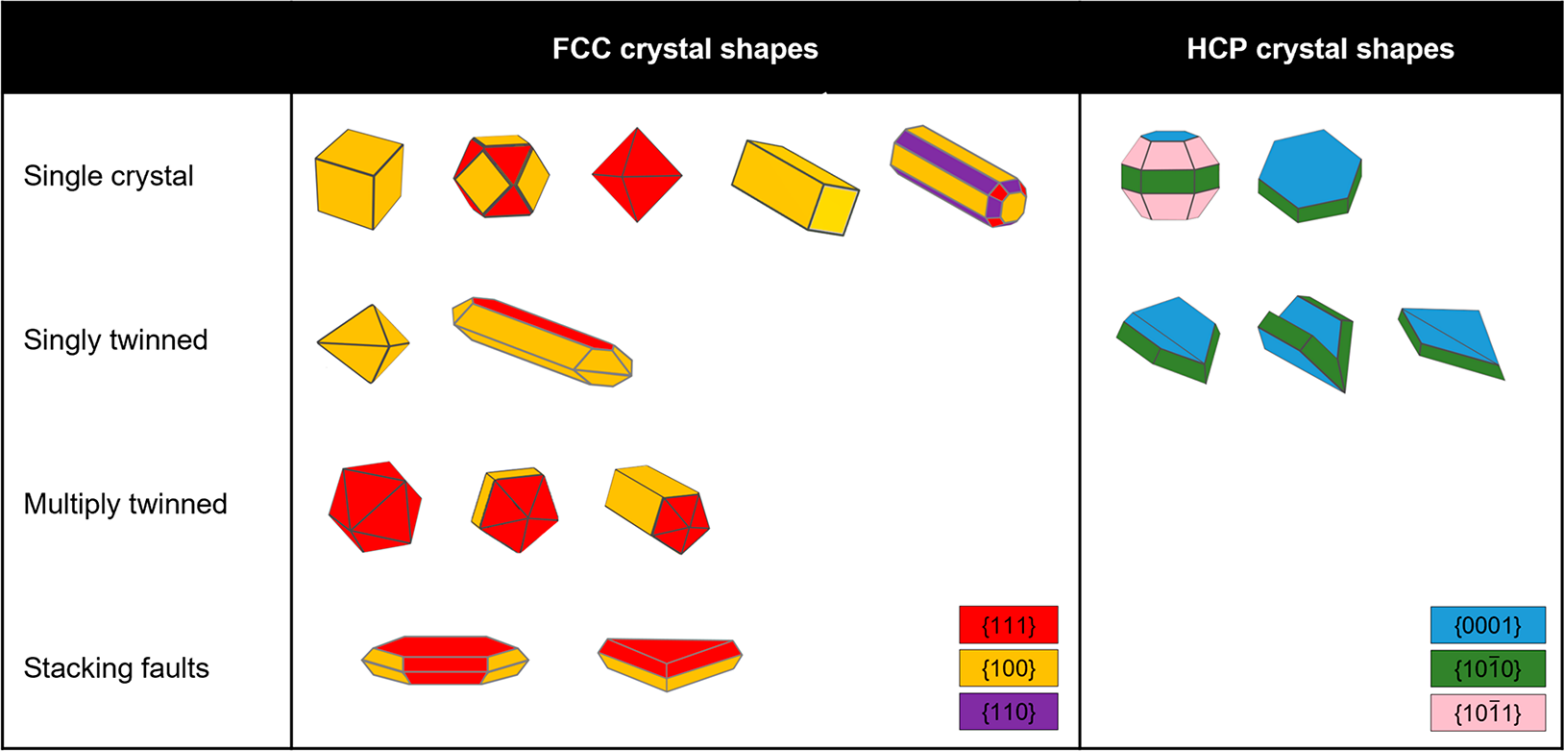
Single crystal and twinned shapes of FCC and HCP NPs. Adapted with permission from ref (94). Copyright 2021 Boukouvala *et al.*

Practically, not all shapes are achievable as they are dictated by the crystallographic structure, twinning, and the relative facet energy (or growth velocity) in the reaction medium. For example, Ag NPs are often cuboctahedral reflecting Ag’s FCC lattice,^65^ while Mg is HCP and often forms hexagonal plates.^76^ Other shapes are obtained by controlling twinning and then facet expression; all FCC metals therefore have access to the same set of shapes,^62,64,95^ mostly convex, some of which are shown in Figure 3. These are better known for Ag and Au, but Al^67−70^ and Cu^71−74^ syntheses are catching up. In crystallizes in a body-centered tetragonal unit cell that is only a slight distortion from FCC.^96,97^ Meanwhile, Mg forms a new set of single crystal and twinned shapes including, unlike cubic systems, concave structures (Figure 3).^76,81^

Twinning determines NP symmetry and thus shape. FCC metals twin along single or multiple (5 or 20) {111} planes; In has equivalent twinning patterns along {101} planes. HCP Mg has recently been shown to twin along a variety of planes including (101̅*x*) with *x* = 1, 2, 3 and (112̅*y*) with *y* = 1, 2, 3, 4.^76^ This leads to a heterogeneous mixture of shapes with each twin plane leading to a different folding angle that influences the LSPR frequency as well as the strength and distribution of the enhanced field (and hence, e.g., the Faraday number). We also expect that Mg’s elongated structures with faceted, sharp tips will lead to enhanced refractive index sensitivity and higher FOM_LSPR_. Such effects have not been published yet due to the heterogeneity of the reaction products, in turn caused by the difficulties associated with controlling the formation of twin planes during NP nucleation. This early reaction control is challenging but likely possible, following in the footsteps of many FCC metals including Au, Ag, and Pd, where the reduction rate of the initial precursor critically affects the twinning observed *via* effects such as kinetic trapping of the initial nuclei and changes in the initial nuclei size.^98−101^ For instance, in Au, the addition of citrate or hexadecyltrimethylammonium bromide (CTAB) produces twinned and single crystalline NPs, respectively, an outcome attributed to the different reaction kinetics in the early stages of the nucleation and reduction.^65,95,102^ The concentration of metal precursor can also manipulate twinning, as was shown for Au.^103^ Postsynthetic etching is also an option.^65,104,105^ Once developed, twinning control in little-explored nanomaterials such as Mg will allow for systematic characterization and harnessing of the various shape-dependent effects discussed above.

NP shape also depends on the relative surface energies (thermodynamic) or growth velocities (kinetic) of facets,^106−108^ leading to a rich library of shapes for a given twinning pattern (Figure 3). Kinetic products predominantly form at low temperatures and short reaction times, and thermodynamic shapes are more closely reached at high temperatures and long reaction times. Growth velocities can be manipulated by surface-coordinating ligands, additives, or solvents; for example, polyvinylpyrrolidone (PVP) passivates Ag {100} facets to form nanocubes, while citrate binds to Ag {111}, yielding octahedra.^107^ Other complementary approaches such as seeded growth and stepwise reactions provide further control owing to the changing environment of reduction, enabling more exotic shapes including nanostars and hierarchical structures.^63,109,110^ Such approaches to controlling size and shape, once again, are well developed for Ag and Au, in development for Al, In, and Cu, and so far nearly inexistent for Mg.

Shape matters, as exemplified by the order of magnitude differences in performance for Au NPs seen above. The addition of HCP NPs to the plasmonic toolbox promises a new library of shapes. These shapes will generate new field distribution and optical behaviors, distinct from those of FCC shapes, and hence, have different performances in field enhancement, sensing, and photothermal conversion. Little is known so far on the synthesis and shape-dependent effects for Mg and even less for more exotic compositions and alloys, such that there are plenty of opportunities for research outputs and new understanding. Ultimately, though, we believe size and shape control will be achieved and that new materials with new crystallographies will offer different geometries that can make a difference in the performance of plasmonic applications.

### Biocompatibility

Nanomedicine is a key application of plasmonic NPs, including in phototherapy, light-controlled drug release, and contrast agents for optical imaging.^111^ Mg offers an alternative approach to biocompatibility than the traditional use of noble metals. Indeed, unlike Au, which is inert but permanent (hence, good for implants), Mg is transient and excretable, qualities ideally suited for drug delivery and the short-term interventions proposed for plasmonic systems.

Au has been widely researched and several clinical trials are ongoing for drug delivery, photothermal therapy, and imaging.^21,22,111,112^ There also have been several studies of Ag NPs *in vitro* although their higher toxicity than Au hinders their use.^113^ In contrast, adults are believed to consume 1–10 mg of Al per day,^114^ while both Cu and Mg are considered essential nutrients with recommended adult daily allowances (RDAs) of 900 μg^115^ and 300–420 mg, respectively.^116^ The body’s ability to process these metals means they can avoid accumulation, which for Au occurs in the liver and spleen and could potentially cause damage.^117^ Such biocompatibility has fostered initial studies that suggest that Cu and Mg NPs are promising for photothermal cancer treatments.^118 ,119^ Similarly, Na and K could present new opportunities since these are also excretable and present in the body in large quantities, if their high reactivity can be controlled, for instance, *via* alloying with another biodegradable metal.

**Figure 4:**
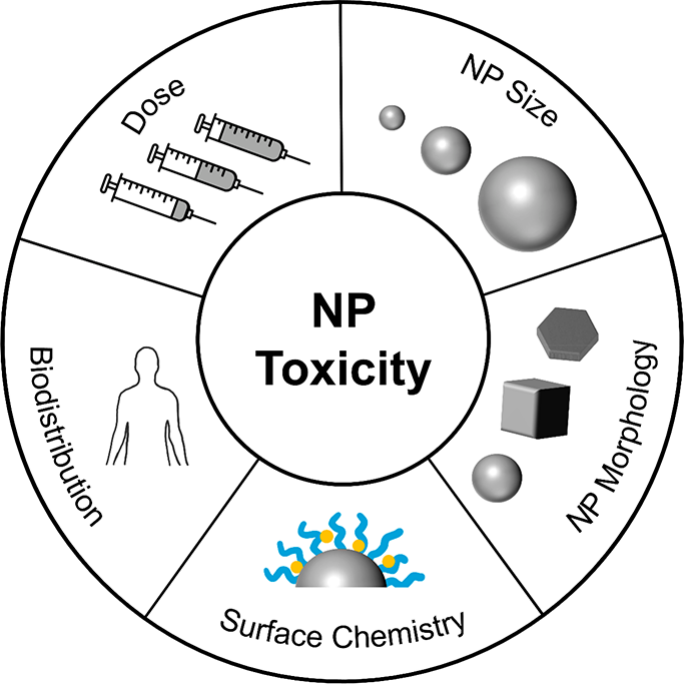
Factors affecting NP toxicity.

However, NP toxicity is dependent not only on the material but also on NP size, morphology, dose, surface chemistry, and biodistribution, as summarized in Figure 4.^21^ NP size modifies toxicity by altering the uptake and accumulation of NPs; *e.g.,* 50 nm Au NPs are likely to accumulate in the liver while 20 nm NPs accumulate in the kidneys.^120^ Shape matters too: Carnovale *et al.* showed that Au nanorods and nanocubes have higher biocompatibility than both prismatic and spherical Au NPs with the same size, dose, and surface chemistry.^121^ Dose is critical as well, such that the “beneficial in low doses, toxic in high doses” behavior of small molecules is also true of NPs. Another key factor of biocompatibility is the NPs’ surface chemistry, which is affected by stabilizing ligands and other chemicals present in the synthesis, including reagents, solvents, impurities, and byproducts. For instance, CTAB, a common ligand in Au syntheses, is cytotoxic even at a low dose and research revealed that it can be removed from Au NP surfaces.^122^ Meanwhile, “green syntheses” using naturally occurring precursors like plant extracts and microorganisms in aqueous mixtures^118^ are emerging to foster biocompatibility for plasmonic NPs, including Au, Ag, and Cu. These effects of size, shape, and surface chemistry are increasingly well-known for Au but have not yet been reported for alternative biocompatible compositions such as Mg, highlighting the infancy of the research field. Yet, much is to be gained from utilizing a biodegradable platform for the many potential nanomedicine applications of plasmonic NPs.

### Oxidation and Reactivity

The reactivity of Cu, Al, Mg, Na, and K underpins their biocompatibility but also creates challenges if they are required to be long-lived in aqueous environments or oxidizing conditions. Reactivity explains why no colloidal syntheses of Na and K are commonplace although approaches to stabilizing fabricated Na structures for SPR exist.^123^ Meanwhile, reactivity complicates the synthesis of Mg and Al, where standard air-free and water-free approaches (glovebox, Schlenk line, *etc.*) are needed to safely obtain a metallic product. On a positive note, though, reactivity can be exploited to forge various multicomponent architectures^124^ as well as create transient structures.^125^ Meanwhile, when needed, managing the surface composition can yield enhanced stability at the cost of a change in the exposed surface chemistry.

The reactivity of Ag toward galvanic replacement by metals of lower oxidation potential, notably Au, has been exploited for decades^126^ as a nanotechnology synthesis tool. Up-and-coming plasmonic metals such as Cu, Al, and Mg can in principle be replaced by a large number of metals owing to their high oxidation potentials.^124,127^ Full replacement provides an approach to obtaining various shapes with some reported structures devoid of the initial metallic template.^128^ Alternatively, controlling the stoichiometry of the replacement reaction leads to decorated structures that retain the plasmonic character of the (now slightly oxidized) metallic core.^124^ Because it does not introduce additional capping ligands or reducing molecules, this approach is appealing for catalysis, green chemistry, and biocompatible structures.

Most plasmonic metals except Au suffer from degradation and often require deliberate protection, which can in some cases also double as a functional element. Shells of a stable compound such as silica, alumina, and iron oxide or of molecules such as polymers and long-chain hydrocarbons are common in nanotechnology.^129^ These have been applied to non-noble metals to enhance their stability, for example, silica on Cu,^130^ polydopamine on Al,^131^ silica on In,^132,133^ and both silica and polydopamine on Mg.^134^ They form a barrier preventing chemical reactions between the core and the surrounding environment. They also enable almost arbitrary functionalization by providing a chemical anchoring mechanism for ligands or shell-like structures capable of adding extrinsic properties such as luminescence, ferromagnetism, light-induced drug delivery, or specific cell labeling.^135−139^ For plasmonic sensing specifically, shells are not always beneficial: they act as a spacer between the metal NPs and the environment, such that field-dependent enhancement effects are reduced, leading to poorer SERS and refractive index sensitivity,^140,141^ although enabling MEF.^142^

Some shells also form spontaneously on reactive metals. While the native oxide layer in Cu can consume the entire NP, metals such as In, Al, and Mg form slow growing (to the point of self-limiting) intrinsic oxide shells, which could in principle be facet dependent. These stabilize the NPs: for instance, we do not observe further growth of Mg oxide over the period of months in solvents and have published numerous results showing its stability in various environments, summarized in ref (6). In most cases, the oxide layer only minimally impacts the LSPRs of the NPs, including a redshift caused by the increased dielectric medium.^6,79,81^ However, the oxide layer dictates the NP’s surface chemistry with little room for maneuverability. On the positive side, however, the oxides formed on Al and Mg are, or can be, of well-known phases with extremely developed chemistry, epitaxy, and substrate effects owing to their use as catalyst supports over the past decades.^143,144^ Further, the thickness of the native oxide shell is not obviously controllable, such that distance-dependent plasmonic enhancement and sensing cannot be easily optimized. Finally, most intrinsic oxide shells fail to protect the NPs from aqueous degradation because of soluble ion formation, such as Mg^2+^ in the case of Mg metal.

While deliberately deposited inorganic or organic shells and spontaneously formed metal oxide shells offer plenty of opportunities for alternative plasmonic metals such as Mg, we believe there is a third, less explored, avenue for stabilization and functionality enhancement: alloying. Small amounts of additives in metals can drive the formation of extremely stable oxides, for example Cr in stainless steel. Further, alloys can stabilize NPs against degradation, as was shown for the decrease of Ag ion leaching in the presence of Au.^56^ Recent work has demonstrated steps toward “stainless Mg”;^55^ however, much remains to be done to adapt these approaches to the nanoscale.

Overall, while oxidation in non-noble metals can be a challenge, stability and functionalization can be secured with intrinsic or extrinsic shells, and reactivity can be used to one’s advantage in the creation of multimetallic architectures.

### Synthetic Costs

**Figure 5:**
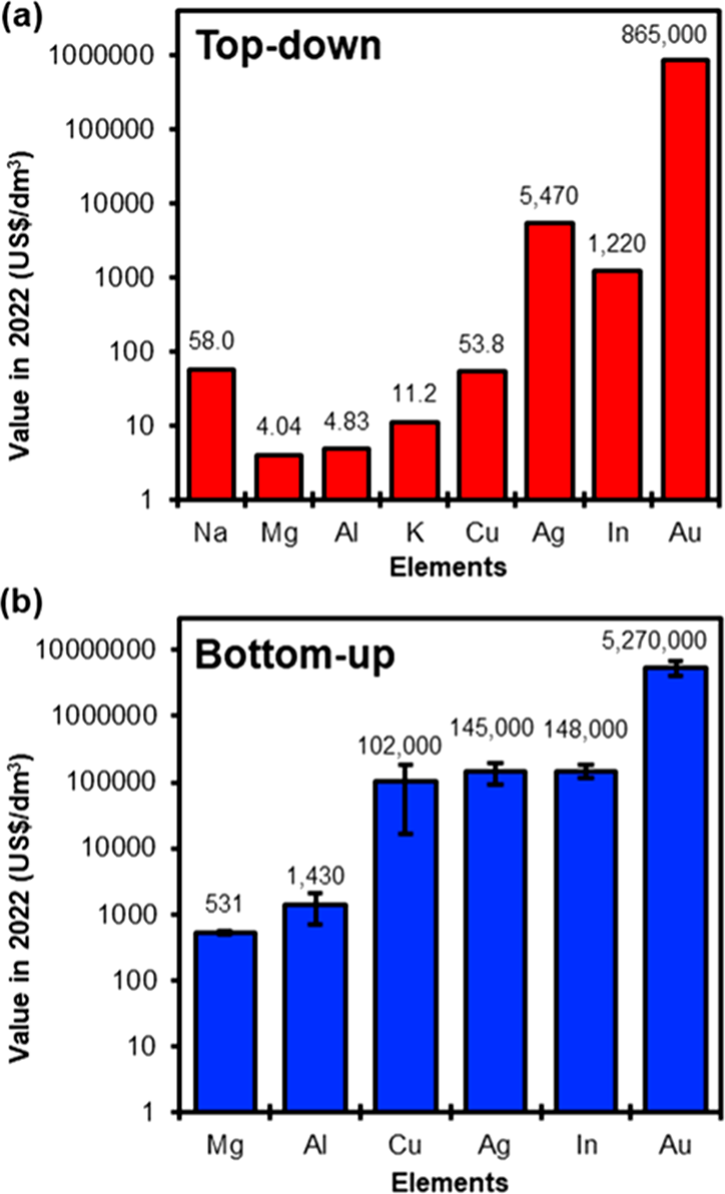
Cost comparison of metal precursors for (a) high-purity bulk metals^147^ and (b) different metal salts used in colloidal synthesis in the literature for Au (HAuCl_4_·3H_2_O, AuBr_3_, AuCl_3_, KAuCl_4_), Ag (AgNO_3_, CH_3_CO_2_Ag, Ag_2_SO_4_), Cu (CuSO_4_·5H_2_O, CuSO_4_, (CH_3_CO_2_)_2_Cu, CuCl_2_, CuCl_2_·5H_2_O), In (InCl_3_, InCl_3_·H_2_O, (CH_3_CO_2_)_3_In), Al (C_2_H_5_N(CH_3_)_2_·AlH_3_, Al(CH_2_CH(CH_3_)_2_)_3_, ((CH_3_)_2_CHCH_2_)_2_AlH, AlCl_3_), and Mg ((CH_3_(CH_2_)_3_)_2_Mg, MgCl_2_); costs are from Sigma-Aldrich. Error bars represent the cost spread across the various salts.

Since noble metals are predominantly used as currency, in arts, and in electronics, the large-scale application of their nanomaterials becomes dependent on their bulk market value. Values are conventionally reported for metals in a nonoxidized state. The market value of plasmonic metals is closely related to their abundance in the earth’s crust (Figure 5a)^145−147^ with some elements facing critical pressures. For instance, multiple sources have predicted impending Au shortages in the next decades if extraction continues at current rates,^146,148^ which could hinder uses of Au NPs, whose metal precursor price already reaches a seven digit figure per cubic decimeter. Meanwhile, In demands have increased dramatically with the use of indium–tin oxide as a transparent electrode in modern electronics, which is reflected in the high price of precursor salts, while for the metals considered, In is the third most expensive metal in bulk, following Au and Ag. Alternative plasmonic metals like Cu, Al, and Mg are orders of magnitude less expensive in bulk and more sustainable in the near future.^149^ However, while Mg and Al metal salts offer prices three or more orders of magnitude lower than Au, Cu prices are comparable to those of both Ag and In. Finally, Na and K metals are also priced competitively; we deliberately omitted precursor costs for the alkali as suitable syntheses have yet to be reported.

The costs of chemical precursors involved in colloidal syntheses also roughly track with abundance, although there is a significant mark-up compared to raw metals, as shown in Figure 5b. This is in part due to the small, research-scale amounts typically purchased at the development stage, and it is likely that mass production would lead to significant discounts, lowering the price of these chemicals to near the price of the precious metals. We will thus use the bulk metal price to assess the financial viability of plasmonic applications below.

Depending on the amount of nanomaterial required by an application, the cost associated with the metal may or may not be limiting. The application requiring the least material is sensing, and even Au is currently commercially viable in this context, for instance in pregnancy tests. Slightly larger quantities are required for therapeutic purposes such as photothermal therapies. For instance, a recent study infused 7.5 mL/kg of a 4.8 mg/mL Au nanoshell solution (nanoshells are 88% Au) per prostate cancer treatment session;^22^ this corresponds to 2.5 g and $120 US worth of metallic Au for a 70 kg patient, not a small expense, yet trifling in the context of cancer therapy in the developed world. It thus appears that raw materials costs are only likely to be limiting in lower value, larger scale contexts. The most prominent of these is plasmon-mediated chemistry, where a plasmonic material is used to absorb and channel sunlight to drive a chemical reaction, often with catalytic surfaces or semiconductors as intermediates. This approach promises greener, fossil-fuel free small molecule industrial reactions. In this context, the plasmonic material is not consumed but is an upfront expense for which one considers a payback time to assess viability. A lower bound on payback time is simply the time required for the reaction to generate enough molecular added value to pay for the plasmonic metal at bulk cost. The evaluation of payback time from prominent examples in the literature reveals that Au plasmonic catalysis is not financially viable. For example, the transformation of benzylalcohol to benzaldehyde with CeO_2_-coated Au nanorods has a payback time of ∼100 years,^150^ while the production of hydrogen from water with TiO_2_-coated Au nanorods has a payback time of ∼100,000 years.^151^ This might seem surprising given the common use of very expensive metals (Pd, Pt) as industrial catalysts; however, plasmonic particles must be at least an order of magnitude larger in diameter than catalytic particles, hence 3 orders of magnitude larger in volume and cost. Beyond Au, the picture is more optimistic. Even Ag is a hundred times cheaper than Au and is a stronger plasmonic performer. For instance, a key paper on the topic turns ethylene into ethylene oxide with Ag at a payback time of ∼1 week.^152^ All the alternative plasmonic metals are cheaper than Ag, some by several orders of magnitude, and so all are likely good candidates for large-scale applications. There is also every reason to look beyond Ag as different metals are suitable for different chemical environments and operating wavelengths. For example, Mg and Al are only appropriate in dry environments, and Ag is poisoned by the presence of sulfur, leading to deactivation (hopefully not before payback time).

Of course, in addition to the cost of raw metals or chemical precursors, both fabrication and synthesis approaches can be labor-intensive and incur a variety of other costs.^153^ For example, lithographic methods require highly trained personnel and significant equipment investment and maintenance costs. Meanwhile, in colloidal approaches, solvents used for synthesis and purification incur waste disposal expenses and ecological issues, and multistep processes for added functionalities increase the costs due to their increased complexity. However, colloidal syntheses can be scaled up, and continuous flow approaches can cut costs by simplifying the processes and their supervision;^154^ ultimately, we believe the raw material costs will set the fundamental limit to financial viability. In this context, the low cost of the emerging earth-abundant plasmonic metals is a key advantage over their noble counterparts, which may be decisive for large-scale applications.

## Conclusion

Over the past decades, NPs sustaining LSPRs have been proposed for a myriad of applications, fueling research and start-up efforts. The field of plasmonics has concurrently expanded and now contemplates the use of alternatives to the well-established Ag and Au. In this Perspective, we discussed the opportunities and challenges offered by these alternative plasmonic metals such as Mg.

The fundamental limit on the performance of a plasmonic system is set by the dielectric function of the material. Here, we saw that all candidate materials, new and old, are rather lossy, leading to broad, damped resonances. The alternative plasmonic metals are not decisively superior but have competitive performance and expand the achievable resonance frequencies. Alloying has so far only demonstrated small improvements, and the minimization of losses in plasmonic materials remains a defining challenge.

NP size matters, as it enables the navigation of the LSPR frequency across the material’s plasmonic range. Size control has been achieved in even the newest plasmonic materials and does not appear to be a limiting factor. Shape matters too, as it dictates the shapes of the resonant modes and their associated field enhancement pattern. Here, alternative metals offer great opportunities, as Mg, in particular, has a different crystal structure than all other candidates, leading to an array of new shapes with promising spiky features. While the controllable synthesis leading to homogeneous products is currently limiting, we expect this hurdle to be overcome, as it has been in the past for Ag and Au and more recently for Al.

However, perhaps the key advantage of the alternative plasmonic materials is that they come with alternative chemistries. For example, Mg is transient rather than accumulating in the body and an essential nutrient rather than a toxin, making Mg an ideal candidate for nanomedicine. Some of the alternative metals are also more reactive, leading to opportunities to create dynamic nanostructures and architectures that use reactivity to assemble multiple functional components. Reactivity also brings the challenge of oxidation, but this is self-limiting in air for some materials and can be prevented entirely with protective shells.

Many of the alternative plasmonic metals are also orders of magnitude cheaper than Au. Yet, it transpires that cost is only a significant limitation for large-scale applications, notably plasmon-mediated catalysis. Here, one must look to Ag or the new alternatives, but in other contexts (sensing, therapy) the amount of NP required is so low that the material cost is not limiting.

We see these materials particularly enabling plasmonic applications in therapy, owing to their extreme biocompatibility, and also in light-driven reactions, owing to their abundance and low cost. Overall, we believe that the emerging plasmonic metals, including Mg, offer competitive plasmonic performance but with radically different crystallography and chemistry and at a fraction of the price.
